# Complete Coding Sequences of Rhinovirus Types A46, A39, C56, and C48

**DOI:** 10.1128/mra.00680-22

**Published:** 2022-10-26

**Authors:** Courtney L. Collins, Temitope O. C. Faleye, Simona Kraberger, Rafaela S. Fontenele, Deborah Adams, Sangeet Adhikari, Helen Sandrolini, Sarah Finnerty, Rolf U. Halden, Matthew Scotch, Arvind Varsani

**Affiliations:** a Biodesign Center for Fundamental and Applied Microbiomics, School of Life Sciences, Center for Evolution and Medicine, Arizona State University, Tempe, Arizona, USA; b Biodesign Center for Environmental Health Engineering, Biodesign Institute, Arizona State University, Tempe, Arizona, USA; c Biodesign Center for Personalized Diagnostics, Biodesign Institute, Arizona State University, Tempe, Arizona, USA; d School of Sustainable Engineering and the Built Environment, Arizona State University, Tempe, Arizona, USA; e Arizona State University Health Services, Arizona State University, Tempe, Arizona, USA; f College of Health Solutions, Arizona State University, Phoenix, Arizona, USA; DOE Joint Genome Institute

## Abstract

We report the coding-complete sequences of rhinovirus types C48, A46, A39, and C56, determined from nasopharyngeal swabs from three individuals with influenza-like symptoms in the United States. One sample showed a coinfection of rhinovirus types A46 and C48.

## ANNOUNCEMENT

Rhinoviruses are positive-sense, single-stranded RNA viruses in the genus *Enterovirus* (family *Picornaviridae*). This genus includes 15 species, among them *Rhinovirus A*, *Rhinovirus B*, and *Rhinovirus C*. Enterovirus genomes encode a large polyprotein, which is autocatalytically cleaved into three smaller proteins (P1 to P3) that are further cleaved into 11 proteins: 4 structural proteins (VP1 to VP4) and 7 nonstructural proteins (2A to 2C and 3A to 3D). In humans, rhinoviruses cause the common cold and trigger approximately 50% of asthma flare-ups and exacerbations of chronic obstructive pulmonary disease (1).

As part of an ongoing surveillance study for respiratory viruses at a university campus, nasopharyngeal swabs were taken from three individuals with influenza-like symptoms at the Arizona State University health clinic in March 2020. Sample collection was part of routine clinical care, which is approved by Arizona State University Institutional Review Board under study identification number STUDY00008985. These samples were negative for seasonal influenza A/B virus via rapid lateral flow immunoassay (Abbott BinaxNOW). To determine the viral etiology of the clinical presentation, RNA was extracted from 200 μl of the resuspended sample using the high pure viral RNA kit (Roche Diagnostics, USA). The RNA was used to prepare libraries using the TruSeq stranded total RNA LT kit with the Ribo-Zero human/mouse/rat kit (Illumina, USA). The 2 × 150-bp libraries were sequenced on a NovaSeq 6000 instrument at Psomagen Inc. (USA). All bioinformatic tools were run with default parameters unless otherwise specified. The demultiplexed reads were trimmed using Trimmomatic v0.39 (2) and *de novo* assembled using metaSPAdes v3.14.0 (3). Viral contigs were identified using blastx (4) and the RefSeq virus protein database (RefSeq release 207). The reads were mapped to the viral contigs using BBMap (5).

**FIG 1:**
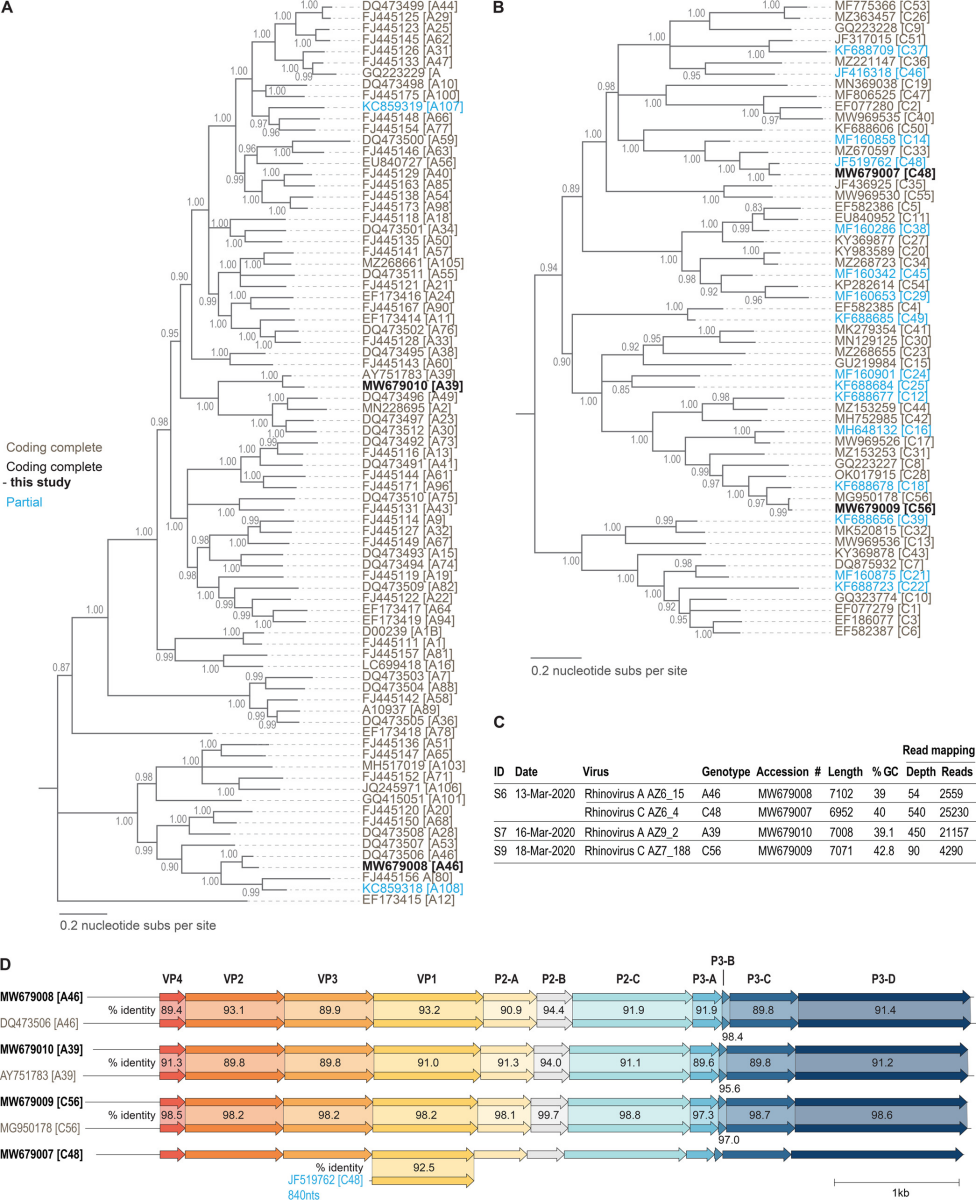
Maximum likelihood phylogeny of the representative sequences of rhinovirus A (A) and rhinovirus C (B), together with the one identified in study, and rooted with sequences of enterovirus D. Genotypes are listed after the accession numbers in square brackets. Branches with posterior aLRT support of <0.8 have been collapsed. (C) Summary of the rhinoviruses identified in this study, including their dates of isolation (day-mo-yr), GenBank accession numbers, read depths (×), and lengths (bp). (D) Pairwise identities of the 11 cleaved protein coding regions within the sequences on the genotypes identified in this study with representatives available in GenBank. Sequence comparison was undertaken using Clinker (10) and nucleotide pairwise identities were calculated using SDT v1.2 (11).

The *de novo* assembled contigs (6,952 nucleotides (nt) to 7,102 nt; coverage depth, 54× to 540×; GC content, 39% to 42.8%) from the three samples had four near-complete genomes (based on the complete coding region for viruses in the genus *Enterovirus*) of rhinoviruses (isolates AZ6_4, AZ6_15, AZ7_188, and AZ9_2). Isolates AZ6_4 and AZ6_15 were identified in the same sample (S6) as a coinfection. Based on blastn analysis, we identified the four rhinovirus sequences as part of the species *Rhinovirus A* and *Rhinovirus C*. Datasets of representative coding-complete or partial sequences of rhinovirus A and C genotypes available at GenBank were assembled into two datasets, together with those from this study and two enterovirus D sequences (GenBank accession numbers D00820 and AY426531) as an outgroup. These two datasets were aligned using MAFFT v7 (6). The two alignments were used to infer maximum likelihood phylogenetic trees using IQ-TREE v2 (7), with GTR+F+I+G4 as the best substitution model. Branches with <0.8 approximate likelihood ratio test (aLRT) support were collapsed using TreeGraph v2 (8). Based on the phylogeny as well as the enterovirus genotyping tool (EGT) (9), isolates AZ6_15 and AZ9_2 were identified as the species *Rhinovirus A*, genotypes A46 and A39, respectively, whereas isolates AZ6_4 and AZ7_188 were identified as the species *Rhinovirus C*, genotypes C48 and C56, respectively (Fig. 1).

In our comparative analysis of the four genotypes, the 11 cleaved protein coding regions share >89% nucleotide identity (Fig. 1), determined using SDT v1.2 (11). For rhinovirus C48, there is only one sequence (GenBank accession number JF519762) of 840 nucleotides (VP1 cleaved protein coding region) that shares 92.5% identity with the sequence we report here (AZ6_4).

### Data availability.

The raw reads and rhinovirus contigs generated in this study have been deposited in the NCBI databases under GenBank accession numbers MW679007, MW679008, MW679009, and MW679010. The raw reads have been deposited under BioProject accession number PRJNA701833 and SRA accession numbers SRR13720058, SRR13720059, and SRR13720060.
